# Ecotoxicity of Nanoparticles

**DOI:** 10.1155/2013/574648

**Published:** 2013-03-24

**Authors:** Sachindri Rana, P. T. Kalaichelvan

**Affiliations:** ^1^Department of Biotechnology, Vels University, P. V. Vaithiyalingam Road, Velan Nagar, Pallavaram, Tamil Nadu 600117, India; ^2^Centre for Advanced Studies in Botany, University of Madras, Guindy Campus, Tamil Nadu 600026, India

## Abstract

Nanotechnology is a science of producing and utilizing nanosized particles that are measured in nanometers. The unique size-dependent properties make the nanoparticles superior and indispensable as they show unusual physical, chemical, and properties such as conductivity, heat transfer, melting temperature, optical properties, and magnetization. Taking the advantages of these singular properties in order to develop new products is the main purpose of nanotechnology, and that is why it is regarded as “the next industrial revolution.” Although nanotechnology is quite a recent discipline, there have already high number of publications which discuss this topic. However, the safety of nanomaterials is of high priority. Whereas toxicity focuses on human beings and aims at protecting individuals, ecotoxicity looks at various trophic organism levels and intend to protect populations and ecosystems. Ecotoxicity includes natural uptake mechanisms and the influence of environmental factors on bioavailability (and thereby on toxicity). The present paper focuses on the ecotoxic effects and mechanisms of nanomaterials on microorganisms, plants, and other organisms including humans.

## 1. Introduction

The nanoparticle and nanotechnology field is a fast-growing research niche [1]. Nanoparticles are particles with at least one dimension less than 100 nm. Nanotechnology is a collective term that implies the capacity to work with materials at a nanometre scale. Nanotechnology thus has potential applications in a wide range of sectors, from energy (production, catalysis, storage), materials (lubricants, abrasives, paints, tires, and sportsware), electronics (chips and screens), optics, and remediation (pollution absorption, water filtering and disinfection), to food (additives and packaging), cosmetics (skin lotions and sun screens), and medicine (diagnostics and drug delivery). This width reflects a diversity of materials that are being or will be used in the different applications [2].

Nanomaterials possess different properties compared to the same material in its coarser or bulk form [3]. Once a material is reduced below 100 nm in size, its components demonstrate unusual features based on quantum mechanics, rather than macroscopic Newtonian mechanics, which influence a variety of material properties such as conductivity, heat transfer, melting temperature, optical properties, and magnetization [4]. Taking the advantages of these singular properties in order to develop new products is the main purpose of nanotechnology, and that is why it is regarded as ‘‘the next industrial revolution” [5, 6].

Nanotechnology is a multidisciplinary field, as it combines the knowledge from different disciplines: chemistry, physics, and biology amongst others [7, 8]. Surface chemistry is also of great importance to the properties of nanoparticles. The decreasing size causes their surface effects to become more significant, due to an increase in the volume fraction of surface atoms, which determines in some instances their special properties [9].

One of the most frequently used procedures involving the preparation of nanoparticles is the use of capping stabilizing agents or surfactants, which help to prevent NPs aggregation and Ostwald ripening [10]. In such cases, stabilizers not only preserve NPs size but also play a crucial role in controlling the shape of the NPs [11].

Several studies exist on the toxicological properties of nanoparticles. Several studies have shown that some of them can pass through the various protective barriers of living organisms. The inhaled nanoparticles can end up in the bloodstream after passing through all the respiratory or gastrointestinal protective mechanisms. They are then distributed in the various organs and accumulate at specific sites. They can travel along the olfactory nerves and penetrate directly into the brain, just as they can pass through cell barriers. Another special feature of nanoparticles is that their toxicity seems to be linked to their surface. Nanoparticles are so tiny that small quantities (expressed in terms of mass), could have major toxic effects, because of their large surface [1]. Soluble nanoparticles toxic effects are linked to their different components, regardless of the particle's initial size. These effects are well known and are not described here. However, the situation is totally different for insoluble or very low solubility nanoparticles [12, 13].

The first feature of nanoparticles is their pulmonary deposition mode, whereby the particles will be deposited throughout the pulmonary system. It has been clearly shown that mucociliary clearance and phagocytosis are well-documented pulmonary clearance mechanisms for micrometric particles [12, 13]. Because of their size, nanometric particles can enter the extrapulmonary organs. This involves migration of solid particles through the epithelial layers and through the nerve endings, along the neuronal axons to the central nervous system. Thus, insoluble nanoparticles pass through the respiratory or gastrointestinal protective mechanisms and are distributed to the various organs throughout the body, including the brain. They are stored in the cells and end up in the bloodstream. These properties are currently being studied extensively in pharmacology, because nanoparticles could be used as vectors to transport drugs to targeted sites in the body [1].

Nanometric particles does not account for the number of particles or the specific surface for a given mass of particles [12]. It is wiser to state the dose according to the characteristics of the nanoparticles (number and surface) instead of their mass. Nanoparticles have a natural tendency to agglomerate, meaning that they group together to form much larger particles [14].

Most industrial applications require unagglomerated particles. Aggregated masses of nanoparticles can be difficult to cleave into their individual primary particles. Thus, despite having small primary nanoparticles, the formation of large aggregates (100 nm) due to particle coalescence can obviate the point of creating a high-surface-area powder [15]. In such a context, producers use different postsynthesis strategies to prevent aggregation or stimulate deaggregation. The nanoparticle surface is modified for the intended application, often by coating the particle with a chemical [16]. These surface changes can have a major impact on nanoparticle toxicity or safety. The manufacturer and the user must consider this reality, which can totally alter the toxicity of a specific product. Little information exists on the impact of these surface properties, except in pharmacology [1].

## 2. Types of Nanoparticles

The types of nanoparticles whose ecotoxic effects are being dealt with in this paper are as follows.

- Fullerenes (grouping Buckminster fullerenes, CNTs, and nanocones and etc.).
- Metal nanoparticles (e.g., elemental Ag, Au, and Fe).
- Oxides (or binary compounds when including carbides, nitrides, etc.), e.g., TiO_2_ and Fe oxides.
- Complex compounds (alloys, composites, nanofluids, etc., consisting of two or more elements), e.g., cobalt and zinc iron oxide.
- Quantum dots (or q-dots).
- Organic polymers (dendrimers, polystyrene, etc.).

## 3. Behaviour of Nanoparticles

Ecotoxicity studies of nanoparticles are scarce and difficult to compare and data from experiments under simplified conditions indicate that some nanoparticles are toxic to a number of organisms, even in very low amounts. This concerns fullerenes, silver nanoparticles, and q-dots, and to a lesser degree carbon nanotubes and nanoparticles of Cu, ZnO, TiO_2_, and SiO_2_. To determine if nanoparticles represent a risk to organisms and the environment, crucial information is lacking regarding mobility, transfer, and uptake as affected by environmental matrices [2]. 

Nanoparticles once released into the environment organisms are likely to be exposed in different ways. Human toxicity studies, with a main focus on workers environments, not only primarily consider dispersion and uptake from air, but also direct uptake through ingestion, dermal exposure and, in some special cases related to medical use, injections. Environmental issues also concern air, as particulate matter dispersed in air will fall out by gravity once they are condensed or aggregated and thus reach a certain size. Both in this and other scenarios for spreading nanoparticles, water may serve as a transport medium and a temporary reservoir for nanoparticles. Yet, the ultimate recipients for any nonvolatile compound or particle spreading in the environment will be sediments and soils [2].

### 3.1. Mobility of Nanoparticles

Nanomaterials end up in the environment, that is, in water, sediments, and soil [17]. Three aspects seem important when assessing the impact of nanoparticles as pollutants ending up in the environment.

1. Mobility (transport and transfer): their ability to move from one place to another (e.g., from a spill site to an uncontaminated site) or from one recipient to another (e.g., from soil to drinking water or food plants).
2. Ecotoxicity: the possible harm that nanoparticles can cause to organisms living in water, sediments, and soils that they enter.
3. Modification: how and to which extent nanoparticles are modified by contact with the environment (and the consequences of such modifications on ecotoxicity and mobility) [2].

A major determinant for particle mobility is the stability of its suspension. If destabilized, a particle suspension will aggregate, which in turn may lead to massive deposition [18].

 Several factors that affect nanoparticle surface potential may destabilize a suspension, including pH changes, increased ion concentration, and dilution or degradation of stabilizing agents (surfactants or coatings) [19]. On the other hand, nanoparticles have large surface area to volume ratios and potentially high sorption capacities for other aqueous species such as natural organic matter [20, 21]. Environmental factors like pH and ionic strength [22] together with the physical-chemical properties and structure and concentration of nanoparticles [23] may determine whether they are bound within or transported out of soils and sediments.

### 3.2. Redox Transformations

Another interesting issue is how redox transformations may influence the transformation and fate of engineered nanoparticles [24]. Redox reactions occur in a wide range of environments and are important for the degradation of organic matter, generation of energy by chemolithotrophic organisms, and the precipitation and dissolution reactions that influence sequestration and mobility of metals. Due to chemical composition, surface charge and high specific surface area, some nanoparticles may have a large capacity to adsorb both inorganic and organic pollutants. One important topic is therefore how nanoparticles may influence mobility, bioavailability, and degradation of potentially harmful compounds in the environment like PAHs, heavy metals, and so forth [2].

### 3.3. Degradability

Nanoparticles are more likely to accumulate in the environment than to disappear. Mineral nanoparticles are more or less prone to weathering and dissolution, and some mineral nanoparticles, like ZnO, are known to dissolve over time when exposed to common environmental conditions. Aggregates of mineral nanoparticles that are formed during preparation in an aqueous environment may be easily broken up mechanically, as has been demonstrated with Fe_2_O_3_ [25]. Organic polymer-based or polymer-like nanoparticles, like starch derivatives, dendrimers, and so forth, are highly biodegradable and are degraded rapidly both in environment and within organisms. Similarly, organic coatings and surfactants that are used to disperse nanoparticles for various applications are easily degradable in the environment [2].

## 4. Ecotoxicity of Nanoparticles

The development of nanotechnologies has introduced important amounts of manufactured nanoparticles into the environment, including those in the ambient air and water. In order to protect human health and wildlife from the potential adverse effects of a broad range of nanomaterials, an increasing number of studies have focused on the assessment of the toxicity of the nanoparticles commonly used in industry [26].

Oxidative stress in terms of ROS (reactive oxygen species) generation is a parameter that is convenient to measure in the context of toxicity and ecotoxicity, because cells respond to oxidative stress by mounting a number of protective responses that can easily be measured as enzymatic or genetic expression responses [27]. Several *in vitro *studies on the toxicity of nanoparticles have shown generation of ROS, for example, by TiO_2_ and fullerenes [28]. On the other hand, some authors have found that nanoparticles, including, for example, fullerenes, may also protect against oxidative stress [29]. This apparent dichotomy underlines the need for research on nanoparticle-cell interactions and mechanistic aspects of nanoparticle metabolism in organisms and specific cells.

Many nanoparticles are photochemically active in the sense that they generate excited electrons when exposed to light (e.g., TiO_2_, ZnO, SiO_2_, and fullerenes). In the presence of oxygen, these electrons can form superoxide radicals by direct electron transfer [30]. Thus, situations where organisms are simultaneously exposed to nanoparticles and light (and in particular UV light which is more energetic than visible light) are of particular concern in an ecotoxicity context.

Ecotoxicity measurements are conducted on different trophic levels including microorganisms, plants, invertebrates, and vertebrates, and test systems have been standardized for some organisms and for some exposure conditions. The standardized protocols specify some physical and physiological conditions (medium composition, age of organisms, etc.) as well as exposure times (e.g., for acute or chronic toxicity) and which endpoints to measure (growth, fecundity, activity of enzymes, expression of genes, etc.). Microorganisms (mainly bacteria, but also fungi, protozoa, and algae) have the advantage that they are ubiquitous and highly diverse (filling a range of habitats and functions), small (permitting miniaturized tests), and with short generation times (permitting rapid tests) [2].

Apart from microorganisms, there are many very useful organisms from different environments that may be used in ecotoxicity testing. It may be interesting to use organisms of different trophic levels (from different steps in a food chain), from primary producers to grazers and predators, as some environmental pollutants may accumulate in the food chain (biomagnification). In soil, organisms may also be selected based on specific modes of exposure (contact, ingestion, and prey preferences), specific habitats (surface, shallow, or deep sub-surface, aerated or anoxic environments, etc.), or specific functions (denitrification, bioperturbation, etc.). In either water or soil, organisms may also be chosen because they are important for carrying out an ecologically process, for example, related to biogeochemical cycling of elements [2].

Ecotoxicity strictly means toxicity to environmental relevant organisms, while the term “bioassay” implies that toxicity or stress caused by a compound has been measured in an environmental matrix pertinent to the habitats where the organism lives in nature. Exposing a fish to nanoparticles in pure water (may be with some added salts) may thus be an ecotoxicity test, while exposing it to nanoparticles in water containing salts, dissolved organic carbon (DOC), and other colloidal materials would constitute a bioassay [2]. The modification of nanoparticles after entering in contact with environmental matrix constituents, like ions, natural colloids, and other charged surfaces is likely not only to affect mobility, aggregation and so forth, but also to modify toxicity characteristics [31].

Like traditional toxicology, ecotoxicology may also use a wide range of physiological and genetic endpoints, but in addition, ecotoxic organisms may be assayed on a functional level, which adds to the complexity of such investigations. Most studies on the adverse effects of nanoparticles have used very pure systems with few possible interactions between nanoparticles and matrix constituents affecting bioavailability. Thus, the conclusions that may be drawn from these results must acknowledge the fact that environmental constituents in water, soil, and sediments (dissolved organic matter, condensed humic substances, clays, etc.) are likely to affect bioavailability and thus toxicity of nanoparticles [2].

Nanoparticles safety doubts have been underlined and their use has come under some scrutiny by both private and public institutions, regarding in particular the possible hazards associated with nanoparticles either deliberately or inadvertently produced [32, 33]. A massive industrial production of nanoparticles in the near future may result in the appearance of both nanoparticles and the waste generated during their production in various environments, yielding the possibility that humans could be exposed to these nanoparticles through inhalation, dermal contact, or ingestion and absorption through the digestive tract. When considering the environmental risks of nanoparticles, a paradox arises when one understands that potentially dangerous nanoparticles also have the potential to produce more environmentally friendly processes, the so called green chemistry, and can be used to deal with environmental contaminants [7, 11, 34, 35]. An example of that is the use of engineered nanoparticles for water treatment and groundwater remediation, which has been proved to be efficient but has also raised concerns for human exposure to nanoparticles contained in the treated water. In order to guarantee the safe use of nanoparticles, some aspects must be taken into account: knowledge, detection, and prevention. An investigation into whether a substance is dangerous or not involves a determination of the material's inherent toxicity, the manner of its interaction with living cells, and the effect of exposure time [36]. It should be noted that the doses or exposure concentrations used in *in vitro *and *in vivo *toxicological studies are most often extraordinarily high in comparison with possible accidental human exposure [16, 32].

Consequently, more research is needed before generalized statements can be made regarding nanoparticles ecotoxicology. Currently, prevention of the escape of nanoparticles to the environment is the best approach under consideration. In this sense, the embedding of nanoparticles into organic or inorganic matrices reduces their mobility and prevents their appearance in the environment [37, 38]. The use of nanocomposites such as these might be the simplest way to increase the safety of nanoparticles. A complimentary approach to ensure the safety of nanoparticles is to use magnetic nanoparticles in their design [39]. Magnetic nanoparticles are of great interest for researchers from a wide range of disciplines due to their useful properties and reaction to a magnetic field [40]. In fact, polymeric materials containing magnetic nanoparticles with certain functionalities (e.g., catalytically active or bactericide) can find numerous technological applications. Their popularity lies in the fact that magnetic nanoparticles can be easily recovered if leakage from the nanocomposite occurs by using simple magnetic traps [41].

## 5. Ecotoxicity of Fullerenes

C_60_ fullerenes have been among the first engineered nanoparticles to be investigated with respect to ecotoxicity, starting with the pioneer work of Eva Oberdörster and colleagues. In Oberdörster's first report on fullerene toxicity [42], she showed that low concentrations of C_60_ (0.5 mg/L) could cause oxidative damage (lipid peroxidation in the brain) and enzyme changes (glutathione reduction in gills) in fish (largemouth bass, *Micropterus salmoides*). C_60_ ecotoxicity is influenced by mode of C_60_ preparation. Bacterial tests of the toxicity of C_60_ fullerenes have shown reduced growth and respiration using *Escherichia coli *and *Bacillus subtilis *[43]. Other studies have shown that bacterial membrane composition and fluidity are sensitive cellular characteristics that are affected by low nC_60_ concentrations [24]. Here, *Pseudomonas putida *decreased its levels of unsaturated fatty acids and increased the proportions of cyclopropane fatty acids in the presence of nC_60_, possibly to protect the bacterial membrane from oxidative stress. *B. subtilis *responded to a low dose of nC_60_ (0.01 mg/L) by significantly increasing the levels of branched fatty acids, and to a high, growth-inhibiting concentration of nC_60_ (0.75 mg/L) by increasing synthesis of monounsaturated fatty acid.

Changes in community structure were indicative of growth impediment for certain species of the indigenous microbial community and demonstrated that even in the presence of a highly complexing medium like soil ecotoxicological effects of nC_60_ may be encountered. The highest concentration of C_60_ was introduced in a dry state without any prior suspension in water. The absence of any ecotoxicological effects could indicate that the bioavailability of C_60_ in the latter experiment was lower in spite of higher total concentrations [2].

Fullerols (1 *μ*M) in combination with UV light have been shown to inactivate viruses (bacteriophages) to a far higher extent (100% increase) than UV light alone [44]. Fullerols have been less toxic than aggregated nonderivatized fullerenes (nC_60_) in a cytotoxicity study on human cell lines [28]. The positively charged C_60_-NH_2_ inhibited growth, reduced substrate uptake, and resulted in damage to cellular structures (cell walls and membranes) in two bacterial species (*E. coli *and *Shewanella oneidensis*) at a concentration of 10 mg/L [45]. In the same study, neutrally charged C_60_ and C_60 _-OH had milder or no negative effects. No toxic effects were observed of fullerol (C_60_OH_16–18_) at 50 mg/L on zebrafish embryos, while nC_60_ at 1.5 mg/L showed among others delayed embryo and larval development and decreased survival and hatching rates [46].

## 6. Ecotoxicity of Carbon Nanotubes

Unprocessed single-walled carbon nanotubes (SWCNT) and double-walled carbon nanotubes were investigated for ecotoxicity to zebrafish (*Danio rerio*) under different salinity conditions. SWCNTs induced a significant hatching delay in Zebrafish embryos at concentrations greater than 120 mg/L. Double-walled carbon nanotubes also induced a hatching delay at concentrations of greater than 240 mg/L, while carbon black did not affect hatching. Embryonic development was not affected at SWCNT concentrations of up to 360 mg/L [47].

In an experiment with Rainbow Trout being exposed to dispersed SWCNT prepared using the surfactant sodium dodecyl sulphate (SDS) and sonication, a dose-dependent rise was observed in ventilation rate and gill pathologies (oedema, altered mucocytes, and hyperplasia) at concentrations between 0.1 and 0.5 mg/L [48]. SWCNTs precipitated on the gill mucus, and the authors concluded that SWCNTs can act as a respiratory toxicant in trout. Copepods ingested purified SWCNTs, but this had no significant effects on mortality, development, or reproduction. Also, exposure to multiple-walled carbon nanotubes (MWCNTs) at concentration higher than 1 mg/L induced a dose-dependent growth inhibition of the cells. A recent study showed that when nanotubes are coated with organic lipids they become more accessible to the water flea *Daphnia magna *[49]. The fleas ingest the materials and strip the lipid layer for food, eventually causing the uncoated nanotubes to block their digestive tracts and kill them. SWCNTs are less toxic to aquatic organisms than fullerenes [50].

## 7. Ecotoxicity of Metal Nanoparticles

The toxicity of a metal is influenced by several factors like solubility, binding specificity to a biological site, and so forth. The toxic effect or heavy metal poisoning is defined as, any functional or morphologic change in the body produced by an ingested, injected, inhaled, or absorbed drug and chemical or biological agent [51]. Metal nanoparticles also exhibit antibacterial activities. Metal nanoparticles exert cytotoxicity depending on the charge at membrane surface. Gram positive cells are less prone to nanotoxic effects due to the presence of thicker peptidoglycan layer compared to Gram negative cells. Nanotoxicity may be attributed to electrostatic interaction between nanoparticles with membrane and their accumulation in cytoplasm [52].

Copper nanoparticles (particles with a biphasic size distribution broadly peaking at 80 and 450 nm) have been investigated for toxicity towards zebrafish (*D. rerio*) and compared to toxicity response towards soluble Cu ions (CuSO_4_) in dechlorinated tap water with a hardness of 142 mg CaCO_3_/L and a pH of 8.2 [53]. In this study, Cu nanoparticles were less toxic than Cu ions. Cu nanoparticles are used as an antimicrobial agent in a similar way as Ag [54, 55].

No data are available on the ecotoxicity of gold nanoparticles. A recent cytotoxicity study on Au nanoparticles ranging in size from 0.8 to 15 nm showed that several human cell types were sensitive to the smallest gold particles (≤1.4 nm) with EC50 values for cell death within 12 h ranging from 30 to 56 *μ*M [56].

Silver nanoparticles are playing a major role in the field of nanotechnology and nanomedicine. Their unique size-dependent properties make these superior and indispensable as they show unusual physical, chemical, and biological properties. Silver nanoparticles have potential antimicrobial activity towards many pathogenic microbes. Along with this antimicrobial activity, silver nanoparticles are showing unacceptable toxic effects on human health and the environment [26]. Ecologists have warned that widespread of such a powerful antimicrobial could have serious negative consequences for bacteria in natural systems if nano-antimicrobials are released in water streams, and so forth. There is also growing evidence that as well as being toxic to bacteria, silver nanoparticles are also highly toxic to mammalian cells [57]. Silver nanoparticles have been shown to damage brain cells [58], liver cells [59], and stem cells. Even prolonged exposure to colloidal silver or silver salt deposits of metallic silver under the skin causes skin diseases like argyria or argyrosis [60]. Even in its bulk form, silver is extremely toxic to fish [61] algae, some plants, fungi [62] crustaceans, and bacteria like nitrogen fixing heterotrophic and soil forming chemolithotrophic bacteria [63].

Silver nanoparticles are mainly produced for antiseptic applications, and they have well-documented bactericidal [64, 65] and cytotoxic effects, including specific effects on mitochondria and generation of ROS [59]. A recent study on uptake of Ag nanoparticles into zebrafish embryos *in vivo* showed an NOEC (no observed effect concentration) as low as 0.19 nM. 

Silver nanoparticles with potential bactericidal activity inhibit soil microbial growth at levels below the concentrations of other heavy metals [3]. It shows toxic effects on human-friendly microbes like heterotrophic (nitrogen-fixing and ammonifying bacteria) and chemolithotrophic bacteria in soil communities. These bacteria also form symbiotic relationships with legumes plants, which provide a major source of fixed nitrogen for both these and other plants. By showing potential toxic effects on denitrifying bacteria, silver disrupts denitrification processes leading to ecosystem disruption [51].

Silver nanoparticles also act as effective antiviral agents viruses like against HIV-1 [66], herpes simplex virus type 1 [67], monkeypox virus [68], influenza virus [69], and Tacaribe virus [70].

Silver nanoparticles prevent osmoregulation in fish as they disrupt the Na^+^, K^+^-ATPase, that helps the active Na^+^ and Cl^−^ uptake [71]. Silver nanoparticles show a potential cytotoxic effect on mammalian germ line stem cells. It was seen here that silver nanoparticles at 10 *μ*g/mL and above concentration showed changes like necrosis and apoptosis of cells [57].

## 8. Ecotoxicity of Nanocomposites

The promise of nanocomposites lies in their multifunctionality, the possibility of realizing unique combinations of properties unachievable with traditional materials [37]. Depending on the nature of the nanophase and the matrix, a wide variety of nanocomposites can be prepared [72]. The idea of using polymer-metal nanocomposites can be advantageous from two different points of view. Firstly, the development of polymer-stabilized metal nanoparticles is considered to be one of the most promising solutions to the issue of nanoparticles stability, by preventing their selfaggregation. Secondly, the use of immobilized nanoparticles reduces the chances of their appearance in the environment [73]. In addition, the incorporation of metal nanoparticles into polymeric matrices can endow the polymer with distinctive properties [74].

The properties of metal-polymer nanocomposites are not determined only by the properties of the metal nanoparticles. The formation of metal nanoparticles within the polymer matrices may strongly modify the polymer morphology, for example, due to the appearance of nanoporosity, which enhances the rate of mass transfer inside the nanocomposites as well as some other structural parameters which are of great importance in their practical applications [75].

New approaches for the complex water treatment are continually being examined. However, it appears to be quite difficult to fulfill all the necessary requirements such as lower overall treatment cost, durability, and high efficiency, higher than the current options for the removal of contaminants from water. Nanotechnology has been identified as a technology that could play an important role in resolving many of the problems involving water purification and quality [36, 76]. The application of metal nanoparticles has been extensively studied for reductive dechlorination of halogenated organic compounds in ground water [77]. One of the most efficient elements is iron nanoparticles as pure monometallic entities or in combination with platinum (bimetallic particles). However, the long-term stability of these nanoparticles can be enhanced by immobilization in a solid support. It is worthy to note that ion exchange materials are widely used for various water treatment processes, mainly to eliminate undesired or toxic ionic impurities such as hardness ions, iron, and heavy metals. The modification of such supports with bactericide metal nanoparticles enables the combination of traditional water treatment with disinfection to eliminate microbiological contaminants. Using this approach, two complementary water treatment steps could be performed with a single material. 

Titanium dioxide nanoparticles seem to have a low toxicity to terrestrial organisms, though few studies are published in this area. TiO_2_ used in sunscreens are nanocomposites where TiO_2_ has been coated with magnesium, silica, or alumina, as well as amphiphilic organics like polydimethyl siloxane (PDMS), and these coatings are modified by ageing. No bioaccumulation of TiO_2_ nanocomposites was observed in the experiment carried out in the anecic earthworm *Lumbricus terrestris* [78].

The stabilization and immobilization of silver nanoparticles in different matrices (nanocomposites) has recently gained great attention from scientists and technologists for two main reasons.

- Immobilization in the matrix can improve the safety of the material (due to the reasonable doubt of their human toxicity).
- The immobilization improves the handling of metal nanoparticles and simplifies their final application [75].

The use of silver nanoparticles containing nanocomposites can also help to solve another important technological problem associated with water treatment, known as biofouling. The biofouling (or biological fouling) is the undesirable accumulation of microorganisms on the surface of water treatment devices and materials such as reverse osmosis membranes, cooling water cycles, and ion exchange resins [72].

 A methodology was developed for the surface modification of commercially available ion exchange materials with core/shell MNPs containing silver shell [79] and a magnetic core [80]. These materials represent the environmentally friendly bactericide nanocomposites suitable for conventional water treatment coupled with reagent-free disinfection. The main advantages of such materials are as follows.

- First, metal nanoparticles are strongly captured inside the polymer matrix that prevents their escape into the medium under treatment.
- Second, the surface distribution of metal nanoparticles within the material provides their contact with the bacteria to be eliminated rapid water disinfection.
- Third, the superparamagnetic nature of metal nanoparticles provides an additional level of the material safety as the use of a simple magnetic trap prevents any postcontamination of treated water with metal nanoparticles leached from the polymer matrix.
- Fourth, the surface location of metal nanoparticles does not essentially influence the main characteristics of the ion exchange material such as the ion exchange capacity and some others, which permits the use of these nanocomposites for complex water treatment, including removal of undesired ionic contaminants and reagent-free disinfection [75].

## 9. Ecotoxicity of Oxide Nanoparticles

The ecotoxicity of TiO_2_ (APS 330 nm), SiO_2_ (APS 205 nm), and ZnO (APS 480 nm) nanoparticles to Gram-positive (*Bacillus subtilis*) and Gram-negative (*Escherichia coli*) bacteria in water suspensions containing citrate and low PO_4_ concentrations was investigated [81]. Antibacterial activity generally increased from SiO_2_ to TiO_2_ to ZnO, and *B. subtilis *was the most sensitive to such effects. Concentration effects were observed for all nanoparticles up to the highest concentrations (5000 mg/L), except for ZnO which showed high and constant inhibition of *B. subtilis *at all concentrations from 10 mg/L. Bacterial growth inhibition was also observed under dark conditions where ROS production was expected to be low. Enhanced toxicity induced by preilluminating TiO_2_ particles shows that the photocatalytic activity of nanoparticles can last for a period of time [2].

Magnetic nanoparticles are of great interest for researchers from a wide range of disciplines, including catalysis, biotechnology/biomedicine, and environmental science and technology [39, 82]. Regarding the materials safety, it is important to notice that due to their low level of toxicity and their good magnetic properties, the use of ferrites is very convenient for biological applications [83]. Core/shell metal nanoparticles of this type can be easily prepared by using IMS technique [38], and the resulting nanocomposites can be applied in catalysis or water treatment [84]. In catalysis, for instance, due to the magnetic properties of metal nanoparticles, the nanocomposites can be easily recovered and reused in sequential catalytic cycles, which is particularly important for metal nanoparticles containing platinum group metals [75].

Phytotoxicity of nanoparticles has been demonstrated for Zn and ZnO as inhibition of seed germination and root growth after 2 h exposure to nanoparticle suspensions in deionized water [85]. Five types of nanoparticles (multiwalled carbon nanotubes, aluminum, alumina, zinc, and zinc oxide) and six plant species (radish, rape, ryegrass, lettuce, corn, and cucumber) were screened. Fifty percent inhibition of root growth (the most sensitive parameter) was observed for nano-Zn and nano-ZnO at approximately 50 mg/L for radish, and about 20 mg/L for rape and ryegrass whereas other nanoparticle-plant combinations showed weaker inhibition.

Results have shown that pure alumina particles significantly reduce the root elongation in all plant species, thus potentially slowing the plants' growth. Since alumina nanoparticles can be released in the atmosphere by exhaust systems, they can also mix with other particles present in the air. Thus the researchers repeated the experiment by loading alumina nanoparticles with phenanthrene (Phen), which is a major constituent of polycyclic aromatic hydrocarbons in the atmosphere and can be absorbed to particular matter in the air. When nanoparticles were loaded with Phen, their phytotoxicity significantly decreased showing no adverse effects on plants' roots. However, the scientists argue that in the field, it is possible that Phen associated to nanoparticles may decompose under UV radiations, thus even the Phen loaded particles in the natural environment may impose adverse effects on plant growth [26].

## 10. Ecotoxicity of Other Nanoparticles

Quantum dots (q-dots) is a diverse group of substances with different composition, size,and coatings. Some q-dots have a strong cytotoxic effect [86] but data on ecotoxicity are lacking. The low amounts necessary for detection of q-dots in medical imaging and stringent policies for recovery of medical waste in hospitals indicate that loss of q-dots to the environment will be very low.

Dendrimers are repeatedly branched organic molecules occasionally used to form hollow cages for transport of drug or other therapeutic agents in nanomedicine. There has been some indication that dendrimers display cytotoxicity [87] and that cationic dendrimers were more cytotoxic and hemolytic than anionic or PEGylated dendrimers [88] as cationic molecules in general can destabilize cell membranes, resulting in cell lysis [89]. No ecotoxicity studies have so far been performed on dendrimers as nanoparticles.

## 11. Environmental Risk Assessment of Nanoparticles

Risk assessment of nanoparticles has started with identification of hazards and exposure routes for humans in a production setting, as this is perhaps the most imminent situation where safety issues have to be resolved to permit other areas of nanotechnology to advance [3, 90]. However, it should also be kept in mind that nanotechnology can have many positive effects also on the environment (sustainable energy, remediation, and more efficient products), and that any possible negative effects and risks should be weighed against these benefits during risk assessment [2].

The first step is to identify and characterise the source of nanoparticles in the environment, the hazards, establish the relationship between dose and response for various endpoints, and then predict the likelihood of exposure. When both dose response and exposure is quantified, they are compared to characterise the relative risk.

### 11.1. Sources of Nanoparticles in the Environment

Sources of NPs can be classified as natural or intentional and unintentional anthropogenic activities. Major natural processes that release NPs in the atmosphere are forest fires, volcanic activities, weathering, formation from clay minerals, soil erosion by wind and water, or dust storms from desert. Atmospheric dust alone is estimated to contain as much as several millions of tons of natural nanoparticel within a year. Naturally occurring ambient nanoparticles are quite heterogeneous in size and can be transported over thousands of kilometres and remain suspended in the air for several days. Accidental releases of nanoparticles into the environment include vehicle exhaust, fuel cells, and different industrial processes. Man-made nanoparticles are unknowingly or purposely released in the environment during various industrial and mechanical processes. The annual release of engineered nanoparticles into the environment cannot be accurately estimated while production volumes are strongly increasing. With the advancement of industrial processes and nanotechnologies, a large number of engineered nanoparticles are been manufactured and it is inevitable that during the use of the related products, nanoparticles are released in the air, water, and soil both intentionally and unintentionally. It also involves techniques like drug delivery, diagnostics, biomedical imaging, ground water remediation, and so forth [91].

### 11.2. Hazard Assessment

In the hazard assessment, the potential of the nanoparticle to cause harm is evaluated. Hazards, such as toxicity and ecotoxicity, can be measured using different endpoints, including physiological, genetic, or functional effects, either acute or chronic. But there may also be other potentially negative environmental effects apart from toxicity that need to be considered in a risk assessment paradigm adapted to nanoparticles like impact on atmospheric/stratospheric processes, stability of soil, effects on bioavailability of mineral nutrients, and so forth [2].

### 11.3. Dose-Response Assessment

Dose-response assessments follow the hazard identification in the risk assessment process. The establishment of dose-response relationships may involve performing experiments in the laboratory or using mathematical models. However, dose-response relationships may not be straight forward for nanoparticles, as dose based on mass concentration may be less relevant than dose based on surface area, and as different nanoparticles preparations may result in differences in surface reactivity, and thus toxicity [2].

### 11.4. Exposure Assessment

The potential for exposure to nanomaterials begins with the production of these materials (as is the case for chemical compounds). Thus, knowledge on quantitative aspects linked to different production steps, purification, functionalization, conditioning, packaging, and transport, as well as losses and waste streams associated with each of these are important. For environmental exposure, it is important to have empirical data or procedures to predict the persistence and mobility in air, soil, and water [2].

### 11.5. Risk Characterisation

Risk characterisation is the final step in the risk assessment procedure, in which the information from the hazard identification, dose response, and exposure steps are considered together to determine and communicate the actual likelihood of risk to exposed populations. The risk is often characterised by comparing the exposure concentrationwith an exposure level that is assumed to have no effect [2].

## 12. Future Research Needs

The most urgent needs for research related to environmental impact of nanoparticles is to establish the degree of environmental mobility and bioavailability. These parameters will decide whether nanoparticles can be taken up and cause harm to various organisms (including plants). This is a pre-requisite for ecological damage as well as effects on public health through entry into drinking water and the human food chain [2]. Research on environmental impact of engineered nanoparticles has started and will face major methodological obstacles regarding detection, characterization, and tracing [92], as well as a dilemma on which nanoparticles to be examined. The first is due to the small size of nanoparticles and the complexity of the environments where it should be studied (water, sediments, soils, and ecosystems), and the latter to the multitude of engineered nanoparticles that exist and their derivatives (e.g., surface modifications of pristine materials) [2].

To study ecotoxicity of nanoparticles, the following should be considered.

- Selection of appropriate terrestrial/aquatic organisms and end points for judging nanoparticles ecotoxicity (or judging fitness for purpose of existing methods).
- Establishing procedures for realistic exposure of organisms to nanoparticles during ecotoxicity measurements.
- Establishing dose-response relationships and toxico-kinetics, including translocation, excretion dynamics, acute versus chronic toxicity, and toxicity mechanisms, (as for basic toxicological studies above).

To study environmental behaviour of nanoparticles, the following should be considered.

- Mobility of nanoparticles in soils, sediments and waste.
- Adsorption/desorption behavior in relation to organic, mineral, and biological components of soil, sediments, and water.

To study metrology of nanoparticles, the following should be considered.

- Development of methods to detect and quantify nanoparticles in air, water, and soils/sediments/waste.
- Testing and adapting existing protocols for analyses of nanoparticles.
- Establishment of standardized requirements for nanoparticles characterization [2].

## 13. Discussion and Conclusion

Nanotechnology is a fast-growing field of activity that will allow development of materials with brand-new properties. The available data indicate that some insoluble nanoparticles can pass through the different protective barriers, be distributed in the body, and accumulate in several organs. Toxic effects have already been documented at the pulmonary, cardiac, reproductive, renal, cutaneous, and cellular levels, while nanoparticles can be distributed throughout the body, including the interior of cells. Significant accumulations have been shown in the lungs, brain, liver, spleen, and bones. The pulmonary route is still the most likely exposure route in the work environment. It is important to realize that the nanoparticle deposition site in the lungs will be affected greatly by nanoparticle dimensions, which can change substantially throughout the production process. Because of their very small size, these particles offer a large contact surface per mass unit. Also, nanoparticles exert cytotoxicity on cells depending on the charge at membrane surface. Nanotoxicity may be attributed to electrostatic interaction between nanoparticles with membrane and their accumulation in cytoplasm. Nanoparticles in plants enter cellular system via roots and stomata, effect transpiration, plant respiration, and photosynthesis, and interfere with translocation of food material. The degree of toxicity is linked to this surface and to the surface properties of these nanoparticles, rather than their mass. A check on the ecotoxicity of nanoparticles is thus very important as it creates a direct link between the adverse effects of nanoparticles and the organisms including microorganisms, plants, and other organisms including humans at various trophic levels. 
